# Cancer cell line-specific protein profiles in extracellular vesicles identified by proteomics

**DOI:** 10.1371/journal.pone.0238591

**Published:** 2020-09-04

**Authors:** Eduarda M. Guerreiro, Reidun Øvstebø, Bernd Thiede, Daniela Elena Costea, Tine M. Søland, Hilde Kanli Galtung

**Affiliations:** 1 Institute of Oral Biology, Faculty of Dentistry, University of Oslo, Oslo, Norway; 2 Department of Medical Biochemistry, Blood Cell Research Group, Oslo University Hospital, Ullevål, Oslo, Norway; 3 Department of Biosciences, University of Oslo, Oslo, Norway; 4 Centre for Cancer Biomarkers CCBio and Gade Laboratory for Pathology, Department of Clinical Medicine, University of Bergen, Bergen, Norway; 5 Department of Pathology, Haukeland University Hospital, Bergen, Norway; 6 Department of Pathology, Oslo University Hospital, Oslo, Norway; Thomas Jefferson University, UNITED STATES

## Abstract

Extracellular vesicles (EVs), are important for intercellular communication in both physiological and pathological processes. To explore the potential of cancer derived EVs as disease biomarkers for diagnosis, monitoring, and treatment decision, it is necessary to thoroughly characterize their biomolecular content. The aim of the study was to characterize and compare the protein content of EVs derived from three different cancer cell lines in search of a specific molecular signature, with emphasis on proteins related to the carcinogenic process. Oral squamous cell carcinoma (OSCC), pancreatic ductal adenocarcinoma (PDAC) and melanoma brain metastasis cell lines were cultured in CELLine AD1000 flasks. EVs were isolated by ultrafiltration and size-exclusion chromatography and characterized. Next, the isolated EVs underwent liquid chromatography-mass spectrometry (LC-MS) analysis for protein identification. Functional enrichment analysis was performed for a more general overview of the biological processes involved. More than 600 different proteins were identified in EVs from each particular cell line. Here, 14%, 10%, and 24% of the identified proteins were unique in OSCC, PDAC, and melanoma vesicles, respectively. A specific protein profile was discovered for each cell line, e.g., EGFR in OSCC, Muc5AC in PDAC, and FN1 in melanoma vesicles. Nevertheless, 25% of all the identified proteins were common to all cell lines. Functional enrichment analysis linked the proteins in each data set to biological processes such as “biological adhesion”, “cell motility”, and “cellular component biogenesis”. EV proteomics discovered cancer-specific protein profiles, with proteins involved in processes promoting tumor progression. In addition, the biological processes associated to the melanoma-derived EVs were distinct from the ones linked to the EVs isolated from OSCC and PDAC. The malignancy specific biomolecular cues in EVs may have potential applications as diagnostic biomarkers and in therapy.

## 1. Introduction

Extracellular vesicles (EVs) are released by cells into the extracellular space and are classified according to their size and biogenesis [1, 2]. Accordingly, EVs with diameters of 30–100 nm and of endosomal origin are defined as exosomes [2]. The EVs which originate by direct outward budding of the cell membrane are named microvesicles (100–1000 nm) and apoptotic bodies (>1000 nm) [2]. EVs are important players in cell-cell communication in health and disease [3] due to their diverse content of biomolecules, such as lipids, nucleic acids, and proteins [4].

EVs are quite abundant in biofluids as they are continuously released by cells [2]. In some diseases, e.g., cancer, the amount of EVs in the biofluids increases [5]. The EVs in the blood of cancer patients are released both by normal and cancer cells, and their number is estimated to be twice of that found in the blood of healthy individuals [6–8]. Oncogenes in cancer-derived EVs can modulate normal host cells, e.g. fibroblasts and macrophages, as well as local cancer cells and metastatic cells [9–11]. In this manner, tumor-derived EVs can contribute to and maintain the Hallmarks of cancer, a panel of acquired abilities of malignant tumors such as cancer cell proliferation, evasion of growth suppressors, resistance to cell death, migration, and invasion as well as modulating normal cells to favor tumor progression by transforming the microenvironment into a more permissive one [12–15].

Additionally, since EVs contain signaling molecules, they are considered be a potential source of diagnostic biomarkers for the prediction of disease, as well as in disease monitoring and treatment decision making [10, 16]. To explore the potential of cancer derived EVs as possible diagnostic and prognostic markers and to expand our understanding of their influence in cell signaling in disease progression, there is a need to isolate EVs from the other components in cell culture supernatant or biofluid of interest (e.g. blood, saliva, urine) [17]. One method for EV separation is combining two size-based separation techniques (ultrafiltration (UF) and size exclusion chromatography (SEC)). These use the size of EVs to separate them from other components that are present in the biofluid or cell culture media. Once isolated, it is of great value to characterize the EV content. Mass spectrometry (MS)-analysis allows for the identification and characterization of proteins in EV samples. However, the enormous amount of data produced by this technique can be quite extensive [18]. Therefore, to extract meaningful information from the extensive list of proteins, Gene Ontology (GO) has become a useful resource, by associating a GO term to each protein or group of proteins in the data set [18]. Overall, GO is a standardized language, or ontology, that describes the function of a gene or a gene product (RNAs or proteins) in three key domains: biological processes, molecular functions, and cellular components [18–20].

In this study, we characterized the protein content of small EVs (diameters under 200 nm), most likely exosomes and small microvesicles. These were isolated by the combination of UF and SEC from cell culture supernatant of three distinct cancer cell lines: oral squamous cell carcinoma (OSCC), pancreatic ductal adenocarcinoma (PDAC), and melanoma, all with poor prognosis [21, 22]. We aimed to provide information on and compare the protein content carried by EVs from these different malignancies. Overall, we attempted to identify an EV specific molecular signature characteristic of each cancer which could be explored for diagnostic work and/or in the prediction of prognosis of these malignancies. In addition, we studied the protein content of the EVs in relation to the carcinogenic processes known as Hallmarks of cancer.

## 2 Methods

### 2.1 Cell culture

Small EVs were isolated from the culture media of human oral squamous cell carcinoma (OSCC) (PE/CA-PJ49/E10; ECACC, Salisbury, UK), pancreatic ductal adenocarcinoma (PDAC) (BxPC3, ATCC, Manassas, USA), and human melanoma brain metastasis (H3; a kind gift from Prof. F. Thonsen, University of Bergen, Norway). The cells were cultured as previously described [23]. In order to reduce the amount of EVs from the FBS, all cell lines where progressively adapted to Advanced media (Gibco, Life Techologies) supplemented with 1% exosome depleted FBS (Gibco, Life Technologies). Briefly, E10, BxPC3, and H3 cells were cultured in complete, conventional media (respectively: Iscove’s Modified Dulbecco’s medium (IMDM; SIGMA), Roswell Park Memorial Institute (RPMI; Gibco, Life Technologies), and Dulbecco’s Modified Eagle Medium (DMEM; Gibco, Life Technologies), all supplemented with 10% FBS). Thereafter, we adapted the cells by sub-culturing them in increasing ratios of Advanced DMEM (E10 and H3) and Advanced RPMI (BxPC3) all supplemented with 1% exosome depleted FBS, 2 mM L-glutamine (Thermo Scientific), and 1X Antibiotic Antimycotic Solution (penicillin, streptomycin, and amphotericin B (PSA), SIGMA). Sub-culturing was carried out during 8 weeks for the E10 and BxPC3 cell lines. Due to a lower proliferation rate of the H3 cells, sub-culturing of this cell line was carried out for a period of 10 weeks. At this point the conventional media had been completely replaced with the Advanced media. Adapted cells were grown in CELLine AD1000 reactor flasks (Argos Technologies), designed for mass production of adherent cells. Cells were kept at 37°C in a 5% CO_2_ atmosphere incubator.

### 2.2 EV isolation

Cell culture supernatants were collected once a week. After collection of the cell culture supernatant, the CELLine AD1000 flasks were washed with warm PBS. Cell culture supernatant and PBS were then pooled. Samples were centrifuged at 4000g for 5 min at room temperature, to remove cell debris and the supernatant was then centrifuged for another 45 min at 15000g, 20°C. Next, 15 ml of the 15000g supernatant was concentrated by UF using Amicon-Ultra 15 Centrifugal Filter Units (Merck Milipore) with a 50 kDa molecular weight cut-off (MWCO), to a final volume of 4 ml. Concentrates were loaded into 30 ml sepharose CL-2B (GE Healthcare Bio-Sciences AB) size-exclusion chromatography (SEC) columns. The concentrated cell culture supernatants were eluted through the columns with the continuous addition of filtered PBS. Eluates were collected by gravity in 20 fractions of 1 ml. For each fraction, protein quantification was determined by spectrophotometry (Absorbance 280nm) in a Nanodrop spectrophotometer (Thermofisher).

Following protein quantification, the EV enriched fractions were identified and pooled. Samples were characterized by nanoparticle tracking analysis (NTA), immunoaffinity capture (Exosome Human CD9 Flow Detection Kit (Dynal^®^, Thermo-Fisher Scientific)), western blot (WB) (anti-CD9 antibody (10626D, Invitrogen, Carlsbad, USA)) and transmission electron microscopy (TEM), as described elsewhere [23]. Briefly, using NTA, the particle concentration and their size were estimated. Samples were analyzed for the presence of classical exosome markers by western blot Isolated EVs were also characterized by immunoaffinity capture for the presence of CD9. Lastly, visualization of the isolated EVs was carried out by TEM.

### 2.3 Proteomics

Following characterization, proteomic analysis was carried out using a total of 20 μg of protein for each of the preparations (n = 3 for each of the cell lines), as described in detail in (S1 File). Briefly, each sample was precipitated and the EV proteins were digested by trypsin. The resulting peptides were analyzed on an Ultimate 3000 RSLCnano-UHPLC system connected to a Q Exactive mass spectrometer (Thermo Fisher Scientific, Bremen, Germany) equipped with a nano-electrospray ion source. The peptide mass/spectra were compared to a reference database (SwissProt database, Human) for peptide identification and the multiple identified peptides are assembled for protein identification. Protein identification and validation, including statistical analysis were carried out using Scaffold_4.8.7 (Proteome Software Inc.) (See S1 File).

### 2.4 Data analysis

Validation of liquid chromatography-mass spectrometry (LC-MS)-based peptide and protein identifications was carried out using Scaffold_4.9.0 (Proteome Software Inc., Portland, OR, USA). Here protein threshold was set at 99.0% to ensure that only proteins with 99% of probability of being present were produced, and the minimum number of peptides was set to 2. Furthermore, the peptide threshold was set at 95%, the minimum probability to determine if a given spectrum identifies a peptide.

Data from the vesicles isolated from E10, BxPC3, and H3 cell culture supernatants were analyzed and extracted individually from Scaffold. All datasets were screened and proteins that were not present in all 3 replicates within a cell line were not included for further analysis.

Comparison of the proteins from the different cell lines was carried out using the Functional Enrichment Analysis Tool (FunRich) (http://www.funrich.org). In addition, the proteins identified in the preparations from E10, BxPC3, and H3 were compared with the Vesiclepedia database (http://microvesicles.org), a web-based open source compendium of biomolecules (proteins, RNAs, lipids, and metabolites) found across all classes of EVs from published and unpublished studies [24]. Proteins from each dataset were ranked according to their average spectral count (SC), a semi-quantitative measure of protein abundance [25]. The top 20 proteins were extracted for a more detailed analysis. Furthermore, proteomes of the EVs from E10, BxPC3 and H3 cell lines were screened for the presence of tumor related proteins previously described in the literature, and their role in the acquisition of cancer characteristics known as Hallmarks of cancer [26–33].

Analysis of the proteomics datasets of the isolated EVs from the different cell lines was carried out using the Database for Annotation, Visualization and Integrated Discovery (DAVID) Bioinformatics Resources 6.8. The proteins were classified according to their gene ontology (GO) term and categorized into functional groups under the term “Biological processes” (GOTERM_BP_FAT). Next, the GO terms were grouped into “Functional annotation clusters”, where the terms are organized into groups based on the premise that genes with similar annotation are functionally related to each other [34]. Functional annotation clusters were ranked according to their enrichment score (ES), indicating the relative importance of each cluster [34, 35]. GO terms, ES and statistical determinants (p-values and False Discovery Rate, FDR) were calculated by the DAVID software.

## 3. Results

### 3.1 EV characterization

**Fig 1:**
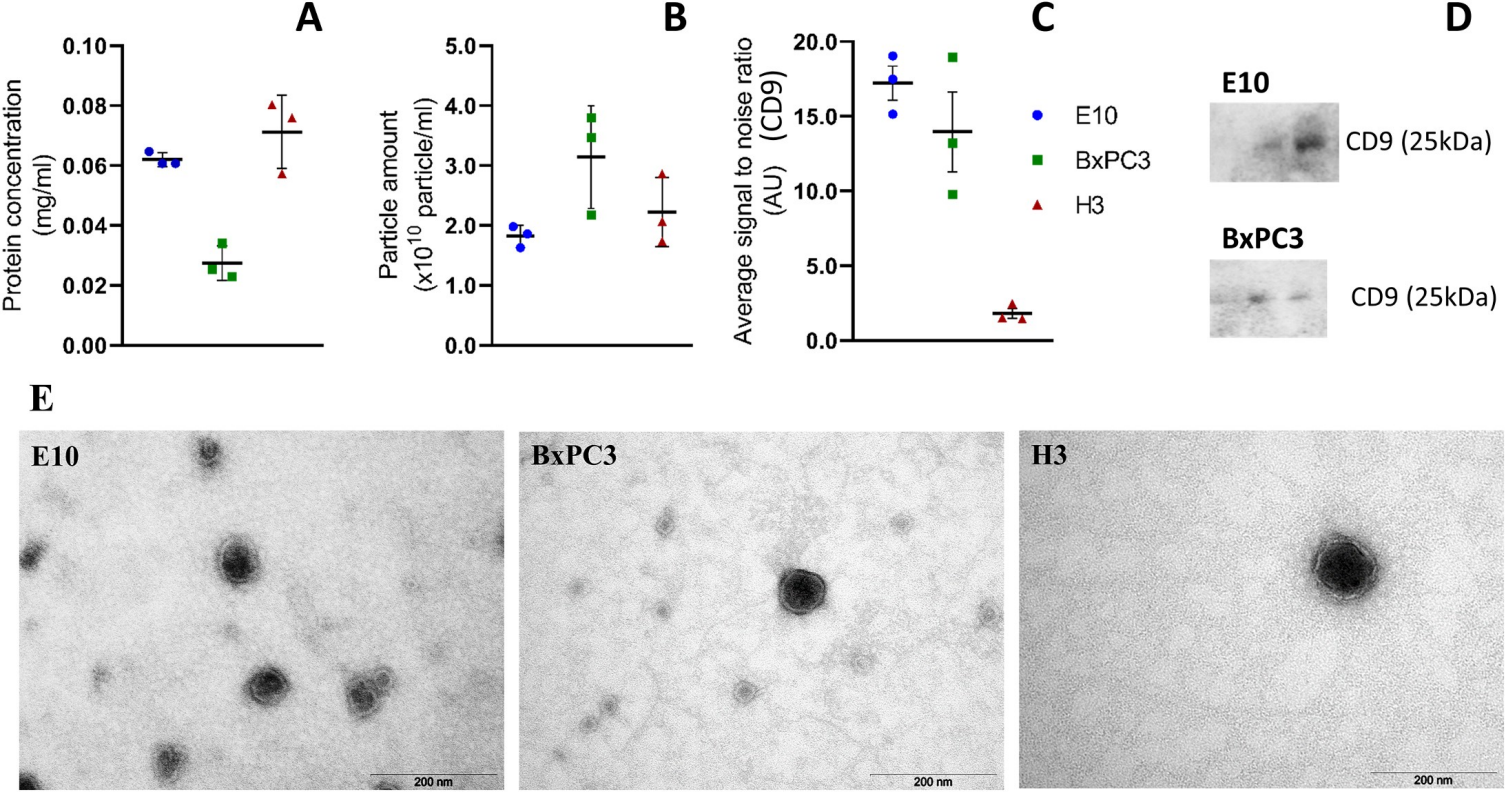
Characterization of the small EVs isolated from the cell culture supernatant of the E10, BxPC3, and H3 cell lines. EVs were studied by protein concentration measurements (A), NTA for particle quantification (B), immunoaffinity capture targeting CD9 (C), and Western blot for the detection of CD9 in three sequential SEC fractions (20 μl per well) (D). Samples were negatively stained with 4% uranyl acetate (aqueous) for analysis by TEM (E). Data republished from Guerreiro *et al* [23].

EVs derived from OSCC, PDAC, and melanoma brain metastasis cell lines were isolated from the cell culture supernatant combining UF and SEC, and were characterized by NTA, WB, immunoaffinity capture, and TEM (Fig 1). Average particle concentration, as determined by NTA, was 1.82x10^10^, 3.14x10^10^, and 2.22x10^10^ particles/ml for E10, BxPC3, and H3, respectively (Fig 1B). Immunoaffinity capture was also used to analyze the presence of CD9 in the small EVs (Fig 1C). E10 and BxPC3 were found to be positive for this marker, while its expression was very low in the vesicles from H3. The EVs were examined for the detection of the markers CD9, CD81, and CD63 by WB. Here only CD9 was detected (Fig 1D), and only in the vesicles derived from the E10 and BxPC3 cell lines. Analysis by TEM showed the presence of EVs with diameters in the range of 60–140 nm, presenting a dark central area surrounded by a lighter peripheral zone corresponding to the membrane of the small EVs (Fig 1E).

The EVs underwent liquid-chromatography mass-spectrometry (LC-MS) analysis for identification and characterization of their protein content.

### 3.2 Overview of EV protein content

Proteomic analysis of the vesicles isolated from cell culture supernatant from the three cell lines identified 678 different proteins from the E10 derived EVs, 618 proteins from the BxPC3 derived EVs, and 762 proteins from H3 derived EVs (Fig 1A). Interestingly, 25% of all the proteins identified were common to all cell lines. Additionally, 7–10% of the total proteins identified were shared by two cell lines. In contrast, 14%, 10%, and 24% proteins were unique in E10, BxPC3, and H3 vesicles, respectively. Furthermore, most of the proteins in the vesicles from the cell lines were already identified and registered in the Vesiclepedia database [36] (Fig 1B), with only a few exceptions (9 proteins in E10, 8 in BxPC3, and 11 in H3; S1 Table). In addition, the 30 proteins most often identified in EVs according to Vesiclepedia, including the vesicle markers CD9, HSPA8, and ENO1, were also found to be present in our preparations. However, CD63 was detected only in the H3 vesicles (S2 Table).

### 3.3 Top 20 proteins of each cell line

The top 20 most highly expressed proteins in the EVs from each of the cell lines are listed in Table 1 together with their known functions. Here, EVs from E10 were found to be enriched in EGFR, ITGB4, NT5E, MYO1C, ATP1A1, and CTNNA1, while enriched proteins in the BxPC3 vesicles were mucins (MUC5AC, MUC5B, MUC16, and MUC2), MVP, COL7A1, PGK1, PLEC, and EEF2. In addition, the most enriched proteins in the EVs from the H3 cell line were HMCN1, HSP8A, APOE, LAMA1, HSP90AB1, CSPG4, PYGB, MYH10, and LAMB1. All the vesicles were also found to be enriched in HSPG2, GAPDH, CLTC, ACTG1, PKM, AGRN, and PTGFRN.

**Table 1:** The 20 most highly enriched proteins in the vesicles isolated from the E10, BxPC3, and H3 cell lines, ranked according to the average spectral count (SC). Functions associated are as described in Uniprot Knowledgebase [37].

E10		BxPC3		H3	
Gene name (SC)	Function	Gene name (SC)	Function	Gene name (SC)	Function
HSPG2 (323)	Integral component of basement membranes; promotes angiogenesis	HSPG2 (495)	Integral component of basement membranes; promotes angiogenesis	HSPG2 (291)	Integral component of basement membranes; promotes angiogenesis
AGRN (188)	Cellular proliferation, migration and oncogenic signaling	MUC5AC (407)	Escape of cancer cells from immunesurveillance/immunosuppressive agent	HMCN1 (276)	Cancer cell invasion and metastasis
EGFR (158)	Promotes tumorigenesis and angiogenesis	AGRN (160)	Cellular proliferation, migration and oncogenic signaling	FASN (162)	Contributor to transformation and tumorigenic potential; cellular growth and proliferation
MYH9 (142)	Cytoskeleton reorganization	MUC5B (135)	Promotes proliferation, migration and invasion of tumor cells	GAPDH (156)	Extracellular vesicle marker
ITGB4 (141)	Tumor cell proliferation, migration and survival; Regulation of keratinocyte polarity and motility	PKM (135)	Tumor cell proliferation and survival	FN1 (137)	Cell adhesion, cell motility, wound healing, and maintenance of cell shape
ACTG1 (128)	Cell motility	LAMA5 (110)	Thought to mediate the attachment, migration and organization of cells	DYNC1H1 (133)	Intracellular trafficking
FN1 (99)	Cell adhesion, cell motility, wound healing, and maintenance of cell shape	MVP (108)	Drug resistance; cell survival and malignant progression	CLTC (129)	Endocytosis and mitosis
MSN (93)	Cell adhesion and mobility	ACTG1 (99)	Cell motility	ACTG1 (115)	Cell motility
LAMA5 (92)	May mediate attachment, migration and organization of cells	CLTC (98)	Endocytosis and mitosis	HSP8A (104)	Tumor growth; antiapoptotic effects
ANXA2 (90)	Intercellular transport, cell division and migration	GAPDH (93)	Extracellular vesicle marker	PKM (104)	Tumor cell proliferation and survival
MUC5AC (90)	Escape of cancer cells from immunesurveillance/immunosuppressive agent	COL7A1 (88)	Promotes cancer progression	APOE (102)	Lipid transport; promotes cancer cell proliferation and survival
CLTC (89)	Endocytosis and mitosis	PTGFRN (84)	Promotion of metastasis	MYH10 (101)	Cell migration
NT5E (89)	Promoter tumor growth	LGALS3BP (82)	Promotes cell proliferation and inhibits apoptosis in cancer cells; promotes angiogenesis	LAMA1 (99)	May mediate attachment, migration and organization of cells
PTGFRN (87)	Promotion of metastasis	MUC16 (80)	Promotes cell growth, tumorigenesis and metastasis	CSPG4 (96)	Migration; epithelial to mesenchymal transition
PKM (83)	Tumor cell proliferation and survival	PGK1 (80)	Promotes invasion and metastasis	AGRN (95)	Cellular proliferation, migration and oncogenic signaling
GAPDH (80)	Extracellular vesicle marker	FASN (72)	Contributor to transformation and tumorogenic potential; cellular growth and proliferation	MYH9 (90)	Cytoskeleton reorganization
MYO1C (76)	Angiogenesis, glucose uptake and progression of cell cycle	PLEC (71)	Involved in tumor growth	PYGB (89)	Drives glycogenolysis in brain
LGALS3BP (73)	Promotes cell proliferation and angiogenesis; inhibits apoptosis in cancer cells	EEF2 (69)	Promotion of cancer cell growth	HSP90AB1 (88)	Molecular chaperone
ATPA1 (69)	Creates the electrochemical gradient of sodium and potassium ions, providing the energy for active transport of various nutrients	ITGB4 (65)	Tumor cell proliferation, migration and survival **R**egulation of keratinocyte polarity and motility	PTGFRN (85)	Promotion of metastasis
CTNNA1 (68)	Cell-adhesion. May be involved in cell differentiation; Invasion suppressor	MUC2 (63)	Tumor suppressive gene	LAMB1 (82)	Proliferation, adhesion, migration, invasion and angiogenesis

Basement membrane-specific heparan sulfate proteoglycan core protein (**HSPG2**); Agrin (**AGRN**); Epidermal growth factor receptor (**EGFR**); Myosin-9 (**MYH9**); Integrin beta-4 (**ITGB4**); Actin, cytoplasmic 2 (**ACTG1**); Fibronectin (**FN1**); Moesin (**MSN**); Laminin subunit alpha-5 (**LAMA5**); Annexin A2 (**ANXA2**); Mucin-5AC (**MUC5AC**); Clathrin heavy chain 1 (**CLTC**); 5'-nucleotidase (**NT5E**); Prostaglandin F2 receptor negative regulator (**PTGFRN**); Pyruvate kinase PKM (**PKM**); Glyceraldehyde-3-phosphate dehydrogenase (**GAPDH**); Unconventional myosin-Ic (**MYO1C**); Galectin-3-binding protein (**LGALS3BP**); Na+/K+ transporting ATPase subunit alpha-1 (**ATP1A1**); Catenin alpha-1 (**CTNNA1**); Mucin-5B (**MUC5B**); Major vault protein (**MVP**); Collagen alpha-1(VII) chain (**COL7A1**); Mucin-16 (**MUC16**); Phosphoglycerate kinase 1 (**PGK1**); Fatty acid synthase (**FASN**); Plectin (**PLEC**); Elongation factor 2 (**EEF2**); Mucin-2 (**MUC2**); Hemicentin-1 (**HMCN1**); Cytoplasmic dynein 1 heavy chain 1 (**DYNC1H1**); Heat shock cognate 71 kDa protein (**HSP8A**); Apolipoprotein E (**APOE**); Laminin subunit alpha-1 (**LAMA1**); Heat shock protein HSP 90-beta (**HSP90AB1**); Chondroitin sulfate proteoglycan 4 (**CSPG4**); Glycogen phosphorylase, brain form (**PYGH**); Myosin-10 (**MYH10**); Laminin subunit beta-1 (**LAMB1**)

Thus, despite the presence of common proteins, the EVs isolated from the different cell lines showed an individual profile.

### 3.4 EV protein content related to the Hallmarks of cancer

When the proteins in our datasets were analyzed in view of the Hallmarks of cancer, we identified proteins with roles in the processes of angiogenesis, inflammation, cell proliferation, migration, immunity, and cell adhesion (Table 2).

**Table 2:** EVs isolated from cell culture supernatants of E10, BxPC3, and H3 contain proteins shown to modulate tumor cells and metastatic environment [26–33] (for more detailed information on protein function and average spectral counts (SC), see S3 Table).

Role \ Cell line	E10	(SC)	BxPC3	(SC)	H3	(SC)
**Angiogenesis**	ITGB1	(68)	MFEG8	(47)	MFEG8	(61)
	TNC	(63)	ITGB1	(19)	TNC	(51)
	ICAM	(55)	ICAM	(9)	ITGB1	(25)
	MFEG8	(47)			ICAM	(2)
**Inflammation**	S100A9	(15)	S100A9	(9)	MIF	(8)
	MIF	(14)	MIF	(7)		
**Proliferation**	EGFR	(158)	ITGB4	(65)	CSPG4	(96)
	ITGB4	(141)	Muc4	(43)	ITGA3	(18)
	ITGA6	(66)	Muc1	(35)	CDC42	(8)
	ITGA3	(59)	ITGA6	(32)	TIMP1	(4)
	ITGA2	(57)	EGFR	(26)	ITGA2	(2)
	CSPG4	(34)	ITGA3	(16)	EGFR	(2)
	ITGA5	(31)	ITGA2	(15)		
	CDC42	(15)	CDC42	(14)		
	ADAM10	(13)	TIMP1	(12)		
	Muc1	(11)	S100A14	(10)		
	S100A14	(9)	ADAM10	(6)		
	TIMP1	(9)	ITGA5	(6)		
	Muc4	(5)	CSPG4	(4)		
**Migration**	ITGB4	(141)	ITGB4	(65)	FN1	(137)
	FN1	(99)	ITGA6	(32)	CSPG4	(96)
	ITGB1	(68)	ITGB1	(19)	TNC	(51)
	ITGA6	(66)	ITGA3	(16)	VIM	(47)
	TNC	(63)	ITGA2	(15)	LOXL3	(40)
	ITGA3	(59)	CDC42	(14)	ITGB1	(25)
	ITGA2	(57)	FN1	(12)	ITGA3	(18)
	CSPG4	(34)	LOXL2	(7)	CDC42	(8)
	ITGA5	(31)	ITGA5	(6)	ITGA2	(2)
	LOXL2	(20)	ADAM10	(6)		
	CDC42	(15)	CSPG4	(4)		
	ADAM10	(13)				
**Immunity**	Muc5AC	(90)	Muc5AC	(407)	PMEL	(26)
	LEG1	(23)	LEG1	(8)	LEG1	(17)
					Muc5AC	(14)
**Adhesion**	ITGB4	(141)	ITGB4	(65)	FN1	(137)
	FN1	(99)	TINAGL1	(58)	ITGB1	(25)
	ITGB1	(68)	THBS1	(53)	ITGA3	(18)
	ITGA6	(66)	ITGA6	(32)	Muc18	(14)
	ITGA3	(59)	ITGB1	(19)	TINAGL1	(2)
	ITGA2	(57)	CD166	(17)	ITGA2	(2)
	TINAGL1	(53)	ITGA3	(16)		
	ITGA5	(31)	ITGA2	(15)		
	ALCAM	(22)	FN1	(12)		
	THBS1	(15)	ITGA5	(6)		
	Muc18	(4)	FN1	(12)		

Integrin beta-1 (**ITGB1**), Tenascin C (**TNC**), S-100 calcium binding protein A9 (**S100A9**), Integrin, alpha 2 (**ITGA2**), Integrin, alpha 3 (**ITGA3**), Integrin, alpha 5 (**ITGA5**), Integrin, alpha 6 (**ITGA6**), Integrin, beta 4 (**ITGB4**), Mucin 1 (**Muc1**), Mucin 4 (**Muc4**), S-100 calcium binding protein A14 (**S100A14**), S-100 calcium binding protein A10 (**ADAM10**), Epidermal growth factor receptor (**EGFR**), Cell division control protein 42 homolog (**CDC42**), Chondroitin sulfate proteoglycan 4 (**CSPG4**), Metalloproteinase inhibitor 1 (**TIMP1**), Fibronectin (**FN1**), Lysyl oxidase homolog 2 (**LOXL2**), Lysyl oxidase homolog 3 (**LOXL3**), Vimentin (**VIM**), Mucin 5AC (**Muc5AC**), Melanocyte protein (**PMEL**), Mucin 18 (**Muc18**), Macrophage migration inhibitory factor (**MIF**), Intercellular adhesion molecule (**ICAM**), Thrombospondin-1 (**THBS1**), CD166 antigen (**ALCAM**), Galectin 1 (**LEG1**), Tubulointerstitial nephritis antigen-like (**TINAGL1**), Tissue factor (**F3**), Lactadhering (**MFGE8**)

The EVs from each of the cell lines demonstrated varied protein profiles related to these processes. For example, the panels of proteins associated with cell proliferation, migration, and adhesion were fairly extensive for both E10 and BxPC3. Of note is the presence of several members of the integrin family (IT), associated with cell proliferation, migration, and adhesion, such as ITGA2, ITGA3, ITGA6, ITGA5, ITGB1, and ITGB4 in the EVs derived from E10 and BxPC3. In contrast, only half of the cell proliferation-related proteins were found in EVs from the H3 cell line, most of them poorly expressed (with SC below 10). The number of proteins related to adhesion were also reduced in the H3 derived EVs. Here ITGB1, ITGA3, and ITGA2 were found in the vesicles from H3, the latter with fairly low SC.

Overall, the protein content of the EVs isolated from the different cell lines carry signals that promote cancer traits. However, the EVs from the H3 melanoma cell line have a more distinct protein signature than the other two, with respect to both the type of proteins as well as their amount present.

### 3.5 EV protein content in relation to biological processes

To better understand the roles and relationships of the proteins in the isolated EVs, Gene Ontology (GO) analysis was performed as described in the Materials and Methods section.

In the dataset of proteins identified in the E10 vesicles, the 8 most enriched biological processes in the functional annotation cluster analysis (Fig 3A) included biological adhesion, cell motility, interspecies interaction between organisms, cellular localization, cellular component organization, response to wounding, cellular component biogenesis, and interaction with host. Interestingly, the same biological processes were identified in the vesicles isolated from BxPC3 (Fig 3B). However, these presented lower enrichment scores than the E10 vesicles. The protein datasets from the EVs isolated from the H3 cell culture supernatant were distinct from the ones of E10 and BxPC3. In H3 (Fig 3C), enriched biological processes included interspecies interaction between organisms, biological adhesion, cellular component biogenesis, RNA metabolic process and protein localization, cellular component organization, cellular response to chemical stimulus, organic acid metabolic process, and extracellular structure organization.

## 4. Discussion

Extracellular vesicles carry complex biomolecular cues and thus, play an important role in the communication between cells. In this study we analyzed and categorized the protein content from EVs derived from oral squamous cell carcinoma (OSCC), pancreatic ductal adenocarcinoma (PDAC), and melanoma brain metastasis cell lines (E10, BxPC3, and H3, respectively), all cancers with poor prognosis for the patient. Isolation of the EVs from the cell culture supernatants, prior to proteomic analysis, was carried out combining UF and SEC. The EVs were characterized by NTA, WB, immunoaffinity capture, and TEM.

### 4.1 Overview of protein content

**Fig 2:**
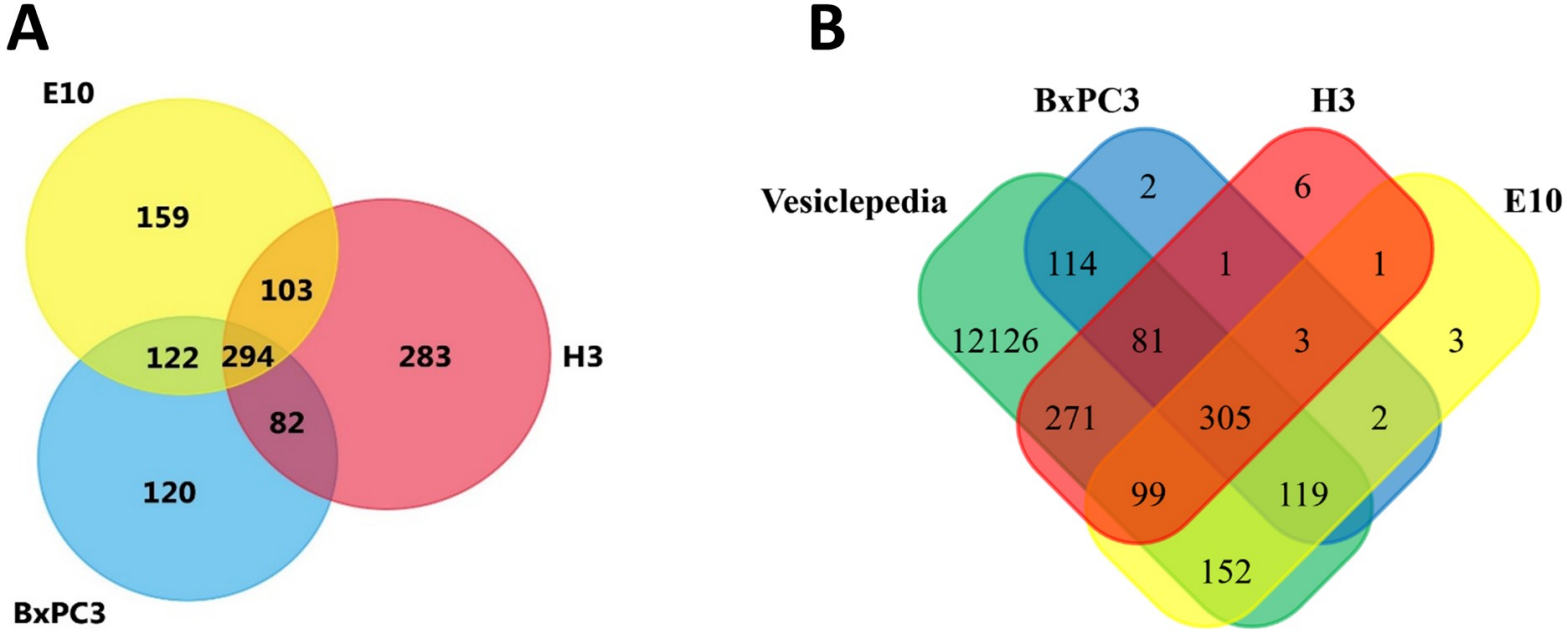
A: Venn diagrams comparing the lists of all the proteins identified in the small EVs derived from OSCC (yellow), PDAC (blue), and melanoma cells (red). B: All proteins from the different datasets were compared with the proteins listed in the Vesiclepedia database (green).

Characterization of the protein content of the isolated vesicles by LC-MS identified a similar number of proteins for all preparations: 678 proteins for E10, 618 for BxPC3, and 762 for H3 (Fig 2A). When comparing the contents of proteins in the different preparations, a number of proteins were found to be unique for each of the cell lines (14%, 10%, and 24% of identified proteins in E10, BxPC3, and melanoma brain metastasis, respectively), suggesting that they have distinct EV profiles. Analysis of the protein datasets produced from the EVs isolated from E10, BxPC3, and H3 was carried out by comparing them with the Vesiclepedia database, a web-based directory of biomolecules (proteins, lipids, RNAs, and metabolites) identified in different classes of EVs [24]. Vesiclepedia is maintained by continuous contribution of the research community as well as manually curated data from published research [24, 36]. Here we found that most of the proteins in the EVs from the different cell lines had previously been reported (Fig 2B). However, the proteins in our samples not yet described in the Vesiclepedia database (S1 Table) were found to be involved in processes such as cell adhesion, apoptosis, regulation of exocytosis, and regulation of vesicular trafficking process. We further investigated the abundance of these proteins in our samples using spectral count (SC), a semi-quantitative measure of protein abundance in proteomic studies [25]. Here we found that the panel of proteins not described previously presented low SCs, most of them SC<5. The work from Lundgren *et al*., indicates that SC of less than five is unrealiable in studies with a small number of replicates [25, 38, 39]. Thus, it is not clear if the absence of these proteins from the database is due to no previous detection or to a low expression level. The 30 most frequently identified proteins in EVs, as reported in Vesiclepedia, were then scrutinized in our dataset (S2 Table). The proteins indexed in the Vesiclepedia list were found to be present in the EV samples from E10, BxPC3 and H3 with the exception of CD63. The reason for the abscence of CD63 in our samples is not clear and should be evaluated in future studies. This is a common extracellular vesicle marker, and its presence has been previously reported in the EVs derived from BxPC3, a cell line used in this study [40]. Nevertheless, CD63 positive vesicles have been described to increase in response to stress [41]. The gradual modification of culture media used in the present study, from the conventional media, supplemented with 10% FBS to the Advanced media, might have lowered the cellular stress, and, therefore, reduced the amount of CD63. Furthermore, the culture conditions may also influence the CD63 levels. Indeed, it has been reported that the presence of CD63 in vesicles derived from different colorectal cancer cell lines depended on whether the cell growth occurred in a 2D versus 3D culture system [42].

### 4.2 The most abundant proteins in EVs isolated from each cell line

The proteins identified in the EVs of each cell line were ranked according to their average SC for further analysis. Due to the large datasets for each of the cell lines, the top 20 most abundant proteins were extracted (Table 1). A characteristic profile began to emerge for each of the cell lines. In the vesicles isolated from E10, EGFR and ITGB4 were found among the top 20. These proteins are known to have relevant roles in the development of OSCC [43]. EGFR is involved in the acquisition of self-sufficient growth-stimulatory signaling, while ITGB4 promotes the tumor ability for invasion and metastasis [43]. In BxPC3, the presence of several types of mucins (MUC5AC, MUC5B, and MUC16) reflected what is described for pancreatic tumors, which are known to overexpress these proteins [44]. For example, while absent in healthy pancreatic tissue, expression levels of MUC16 increase in moderately to poorly differentiated cancers, as is the case of the BxPC3 cell line used [44, 45]. The roles mucins hold in the progression of pancreatic cancer include regulation of the detachment of the cells from the primary tumor, invasion, and enhancement of the survival of detached cells [46–50]. In addition, these molecules also promote adhesion at metastatic sites [50–52]. Interestingly, MUC2 was found in our material. This is a tumor suppressor protein that is down regulated in pancreatic cancer [53]. Its presence among the proteins identified in the EVs derived from the BxPC3 cell line can be explained by the fact that the cells themselves of this particular cell line expresses MUC2 [54, 55]. For the top 20 highly expressed proteins in the EVs isolated from the H3 cell line, LAMA1 and LAMB1, as well as CSPG4, stand out. Here, LAMA1 and LAMB1 have been identified as potential markers for cutaneous melanoma metastasis as these proteins are involved in the modulation of cell adhesion and migration [37, 56, 57]. Also implicated in those latter processes is CSPG4 [37]. Furthermore, CSPG4 is involved in the activation of survival and growth pathways [58–60]. In addition to the proteins that are characteristic for each particular cell line, the proteomic results also identified proteins shared by all the cell lines. Those proteins were HSPG2, GAPDH, CLTC, ACTG1, PKM, AGRN, and PTGFRN. The specific role of HSPG2 and AGRN in EVs is not yet clear although they are known to be components of the extracellular matrix (ECM), the molecular scaffold for tissue organization [37, 61]. Together, this could support the hypothesis that EVs play a functional and structural role in ECM by participating in matrix organization by regulating cell migration or establishing molecular networks within the ECM [62]. In addition, GAPDH, a so-called housekeeping protein that is often identified in the proteome of EVs, does not have a clear role in EVs. It has been hypothesized that the presence of this protein, as well as other glycolytic enzymes, is related to the maintenance of the EVs’ own energy that may be required for the uptake of cancer derived EVs by cells in the tumor microenvironment [63]. The remaining proteins are involved in the maintenance of the membrane structure (ACTG1 and PTGFRN), the uptake of EVs by endocytosis (CLTC), and the release of exosomes (PKM), in particular from cancer cells [64–67].

In summary, our results highlight that the EVs isolated from each of the different cancer cell lines have distinct protein profiles. Cell line-specific profiles already begins to emerge when comparing the 20 most adundant proteins. Here, proteins that seem to provide tumors with signals that promote its progression are found among EV-related proteins. These EV related proteins are present in the datasets from all three cell lines.

### 4.3 Survey of EV protein contribution to cancer characteristics

The panel of proteins detected in the EVs from OSCC, PDAC, and melanoma cell lines (E10, BxPC3, and H3, respectively) indicates that these vesicles can play roles in angiogenesis, inflammation, proliferation, migration, as well as in modulation of immunity, all representing Hallmarks of cancer. Within this selection of proteins (Table 2), there were noticeable differences among the cell lines. For example, there were more proteins associated with cell proliferation in E10 and BxPC3 compared to H3. This can be due to the proliferative characteristics of each of the cell lines, with E10 presenting the highest growth rate and H3 the lowest. In addition, although EVs from the three cell lines expressed many of the same proteins, their amounts differed, as demonstrated by EGFR, Muc5AC, and FN1. EGFR was quite abundant in the EVs from the E10 cell line, but its expression levels were at a much reduced level in BxPC3. EGFR levels in H3 were barely detectable. The expression levels of EGFR in the E10 derived EVs may reflect the high expression of EGFR in OSCC tissue, as shown by immunohistochemistry (IHC), and its abundance in the saliva of OSCC patients, compared to controls [68, 69]. EGFR has been detected by IHC in PDAC tissues as well as in the EVs derived from PDAC cell lines, including BxPC3 [70, 71]. Studies on the transport of EGFR by tumor derived EVs revealed that the transported EGFR will localize in the membrane of the recipient cells, thus preparing distant sites for metastasis [72]. The presence of EGFR in the E10 and BxPC3 derived EVs may suggest that the same mechanism is at play with the exchange of growth factor receptors between the cells of the primary tumors, as well as between the tumor cells and other cells in the tumor microenvironment and even cells in distant sites. The significance of the difference we found in EGFR expression levels in the E10 and BxPC3 derived EVs needs to be further investigated. The very low expression levels of EGFR in the H3 derived EVs could be in line with what has been described in other studies where the presence of EGFR appears to only be associated with a subset of melanomas [73]. It has also been observed that EGFR expression in different melanoma cell lines and their EVs may vary [74].

In contrast, Muc5AC was found to be highly enriched in the BxPC3 derived EVs, while present in much lower amounts in the EVs from the other two cell lines. Muc5AC is a protein that stimulates angiogenesis in PDAC and might also suppress the immune response towards the tumor [75]. In patients, Muc5AC is known to be up-regulated in PDAC tissues as well as in blood [76]. In this manner, our findings are in line with these previous studies. We could not find published work describing the expression of this protein in OSCC and melanoma. However, other members of the Muc family, Muc1 and Muc4, are associated with high aggressiveness of OSCC [77, 78].

Lastly, FN1 was abundant in the H3 derived EVs but detected in a smaller amount in the E10 derived EVs. Furthermore, a dramatic drop in the FN1 content was found in the BxPC3 derived EVs. FN1 plays a role in cell adhesion, migration, and differentiation [32]. Furthermore, FN1 is highly expressed both in metastatic melanoma and OSCC cells [79, 80]. The expression levels of FN1 in H3 and E10 derived EVs reflect those results. H3 is a cell line established from a melanoma brain metastasis and E10 from a primary OSCC. In contrast to our results, analysis of FN1 by IHC in PDAC tissues has demonstrated that this protein is abundant in the tumor microenvironment [81]. Whether the very low amount of FN1 in our BxPC3 derived EVs could be due to a selective protein loading into the EVs requires further investigation.

Differences among the cell lines were also noticeable regarding members of the integrin family. The members of this family of cell adhesion receptors are relevant in cancer processes such as cell migration and invasion [82, 83]. In this study, a higher number of different integrins was detected in the EVs derived from E10 and BxPC3 cell lines, in comparison to H3. The low number of integrin members in the H3 derived EVs may be due to the cell line being generated from a melanoma brain metastasis. According to Hoshino *et al* the levels of integrin expression in exosomes derived from melanoma brain metastasis are down regulated [84]. Moreover, proteins identified in our datasets were also detected in studies on biofluids from OSCC, PDAC and melanoma patients. Some proteins present in the OSCC cell line derived EVs in the present study were identified by Winck *et al* in saliva from OSCC patients (e.g., complement C3) [85]. Similarly, EVs derived from the PDAC and melanoma cell lines included proteins (e.g., ALCAM and galectin-3, respectively) previously reported to be up-regulated in the serum of patients with PDAC and melanoma, respectively [86, 87]. This is an interesting finding and suggests a benefit of parallel studies on cancer cell lines and saliva/serum from cancer patients. This approach might contribute to the identification of possible prognostic markers in saliva/serum of patients.

Overall, the EV content reflects the characteristics of each cell line. Additionally, the cell line specific EV protein combinations point to the cellular phenotypical characteristics described as Hallmarks of cancer. Furthermore, the proteins identified have been detected in clinical cancer tissues in other studies, indicating that these may be relevant as diagnostic biomarkers for these cancers.

### 4.4 Potential roles of EV proteins in relation to biological processes

**Fig 3:**
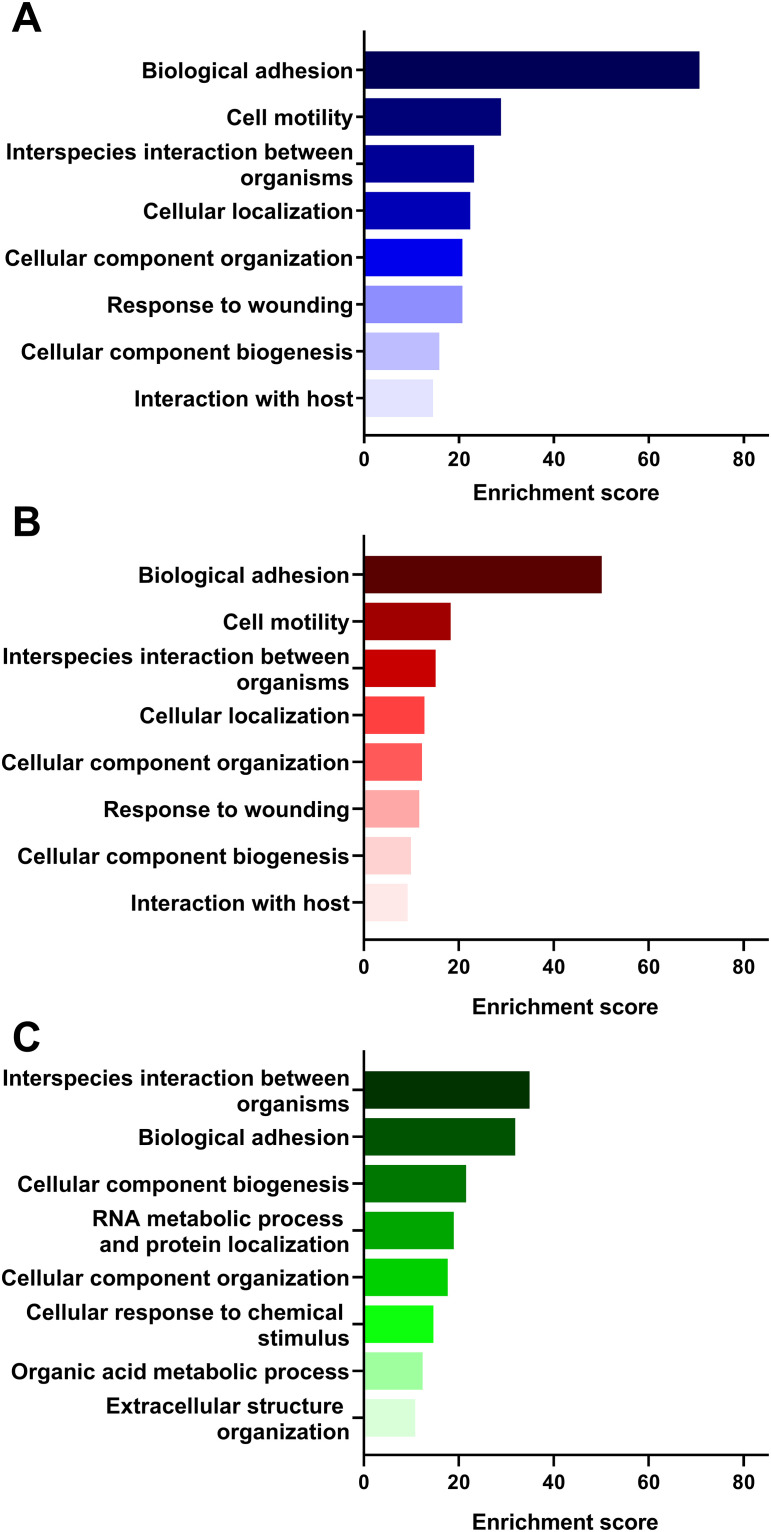
Gene Ontology (GO) functional enrichment analysis of the biological processes (GOTERM_BP_FAT) of the identified proteins in the EVs isolated from the cell culture supernatant of E10 (A), BxPC3 (B), and H3 (C). Biological processes for each cell line were ranked according to their enrichment score, revealing which processes were more relevant within each data set.

Functional enrichment analysis was performed using DAVID annotation clustering. Here, we identified the most enriched biological processes regulated by the hundreds of proteins identified in the EVs from each cell line (Fig 3). We found that the biological processes linked to the H3 derived EVs were quite different from the ones associated with either E10 or BxPC3. The most enriched biological processes unique for the H3 derived EVs were “RNA metabolic processes and protein localization”, “cellular response to chemical stimulus”, “organic acid metabolic process”, and “extracellular structure organization”. In contrast, “cell motility”, “cellular localization”, “response to wounding”, and “interaction with host” were the processes enriched in E10 and BxPC3. Nevertheless, several of these biological processes are found to be associated with cancer. For example, in H3 the “RNA metabolic processes and protein localization” and “cellular response to stimulus” highlight a possible dysregulation of RNA processing which may drive tumor progression [88]. “Cell motility” and “response to wounding” in E10 and BxPC3 are important for cancer cell invasion of surrounding tissues and in establishing metastasis [89, 90]. Whether the differences observed between H3 and E10/BxPC3 are a consequence of the first being a cell line derived from a metastasized melanoma, while both E10 and BxPC3 are derived from primary tumors, is not clear. However, there is evidence that a metastasis possesses a gene expression program distinct from the primary tumor that have allowed for the cancer cells to disseminate [91], which in turn may be reflected in the EV signaling.

The EVs derived from the three cancer cell lines also had some biological processes in common. “Interspecies interaction between organisms”, related to viral reproduction is one example. This process reflects the similarity of EVs and viruses even though EV biogenesis is a naturally occurring biological process while viruses hijack the cellular machinery for its propagation [92]. In addition, all three cell lines had in common the biological processes of “cellular component organization” and “cellular component biogenesis”, which are related to production, assembly, and localization of elements of a cell.

Together, these findings reveal that the EVs analyzed in our study contain proteins that can regulate biological processes towards a cancer phenotype, in a manner that is characteristic for each of the cell lines.

## 5. Concluding remarks

In conclusion, in the present study we used LC-MS to characterize the protein content of small EVs derived from OSCC, PDAC, and melanoma brain metastasis cell lines. More than 600 proteins were identified in the EVs of each cell line. Analysis of the datasets revealed cell-specific protein panels. In short, EVs derived from E10 had a high expression of EGFR, ITGB4, and NT5E, the EVs isolated from BxPC3 were enriched in mucins (MUC5A, MUC5B, MUC16, and MUC2), and the most abundant proteins in the EVs from the H3 cell line included HMCN1, HSP8A, and APOE. Furthermore, screening of the protein datasets for proteins involved in the Hallmarks of cancer also exposed cell-specific protein panels. Here, proteins associated with the promotion of processes such as angiogenesis, proliferation, migration, or adhesion were found to be present in the EVs derived from all three cell lines. This is relevant due to the role of EVs in the modulation of the cells in the tumor microenvironment (e.g. fibroblasts, macrophages and the cancer cells themselves) into a more permissive setting for tumor progression. The list of proteins involved, for example, in cell proliferation was quite extensive in E10 and BxPC3 in comparison to H3 where only half of the proteins were found and most of them poorly expressed. Functional enrichment analysis by gene ontology (GO) was carried out to extract a broader biological significance. Here, processes such as “biological adhesion”, “cell motility”, and “cellular component biogenesis”, all associated with aspects of cancer, were found. However, the processes identified from the proteins from the H3 derived EVs were distinct from the ones in the datasets from E10 and BxPC3, revealing an underlying difference between the cell lines. This study focuses on the protein content of small EVs, and does not explore other classes of biomolecules such as RNAs. Nevertheless, the protein lists generated and analyzed here may provide important information regarding the complex biomolecular cues EVs carry. In a clinical setting, biofluids from cancer patients will include a higher number of cancer-derived EVs in addition to non-cancer-related EVs. This will complicate the collection and examination of spacific caner-derived EVs. Hence, we believe it is important to start with a broad analytical approach that may assist in pinpointing the cancer-derived EVs in biofluids.

## Supporting information

S1 Table (PDF)

S2 Table (PDF)

S3 Table (PDF)

S1 File (PDF)
