# Supplementary material for: Cancer cell line-specific protein profiles in extracellular vesicles identified by proteomics
Source: PLoS One. 2020 Sep 4;15(9):e0238591. doi: 10.1371/journal.pone.0238591 (PMC7473518; doi:10.1371/journal.pone.0238591)
Supplement: S1 Table — (PDF) [file pone.0238591.s001.pdf]

Supplementary Table 1

List of the proteins identified in the vesicle preparations from the different cell lines not yet reported in the Vesiclespedia database, their known function as described in UniProt, and their relative abundance (Spectral counts, SC)

Gene name	Protein	Function	Cell lines		
			E10	BxPC3	H3
			SC	SC	SC
EVA1B	**Protein eva-1 homolog B**	Unknown	3		
PI3	**Elafin**	elastase-specific inhibitor	2,7		
ADGRE5 (CD97)	**CD97 antigen**	Receptor; potentially involved in adhesion and signaling processes after leukocyte activation.	2,3		
ERO1A	**ERO1-like protein alpha**	Involved in disulfide bond formation in the endoplasmic reticulum; also regulates calcium release from the ER		7,7	
NAPRT	**Nicotinate phosphoribosyltransferase**	Helps prevent cellular oxidative stress via its role in NAD biosynthesis		6	
TIGAR	**Fructose-2,6-bisphosphatase TIGAR**	Regulates glycolysis and protects against oxidative stress			3,7
ACSS1	**Acetyl-coenzyme A synthetase 2-like, mitochondrial**	Essential for energy expenditure under ketogenic conditions (By similarity). Converts acetate to acetyl-CoA to produce ATP and CO_2.			2,3
GCN1	**eIF-2-alpha kinase activator GCN1**	Reprogramming of amino acid biosynthetic gene expression to alleviate nutrient depletion			2
NARS2	**Probable asparagine--tRNA ligase, mitochondrial**	Putative member of enzymes involved in protein biosynthesis			2
ACOT2	**Acyl-coenzyme A thioesterase 2, mitochondrial**	Fatty acid metabolism			2
P3H1	**Prolyl 3-hydroxylase 1**	Possible role as a chaperone in the synthesis of collagens; May be involved in the secretory pathway of cells; has growth suppressive activity in fibroblasts			1,7
TLDC1	**TLD domain-containing protein 1**	Activation of alternative mTOR signaling in the regulation of cell proliferation and migration	9,7	3	
COLGALT1 (GT251)	**Procollagen galactosyltransferase 1**	Facilitates the formation of collagen triple helix	3,7	2	
ADGRL2 (AGRL2)	**Adhesion G protein-coupled receptor L2**	Calcium-independent receptor; probably implicated in the regulation of exocytosis	3		6,3
SNU13 (NH2L1)	**NHP2-like protein 1**	Involved in pre-mRNA splicing as component of the spliceosome		2,3	2,3
ADGRG1 (GPR56)	**Adhesion G-protein coupled receptor G1**	Receptor involved in cell adhesion and probably in cell-cell interactions. Plays a critical role in cancer progression by inhibiting angiogenesis	5	4,7	15
RTCB	**tRNA-splicing ligase RtcB homolog**	Catalytic subunit of the tRNA-splicing ligase complex; may act as an RNA ligase with broad substrate specificity	4	4	5
MVB12A	**Multivesicular body subunit 12A**	Component of the ESCRT-I complex, a regulator of vesicular trafficking process. Required for the sorting of endocytic ubiquitinated cargos into multivesicular bodies.	3	5,3	3,3
