# Supplementary material for: Cancer cell line-specific protein profiles in extracellular vesicles identified by proteomics
Source: PLoS One. 2020 Sep 4;15(9):e0238591. doi: 10.1371/journal.pone.0238591 (PMC7473518; doi:10.1371/journal.pone.0238591)
Supplement: S2 Table — (PDF) [file pone.0238591.s002.pdf]

Supplementary Table 2:

List of the 30 most commonly reported proteins in EV studies, as described in Vesiclepedia database, present in the vesicles isolated from the supernatant of E10, BxPC3 and H3 cell lines and their relative abundance (Spectral counts, SC)

		Average SC		
		Cell Line		
Gene name	Protein description	E10	BxPC3	H3
PDCD6IP	Programmed cell death 6-interacting protein	**51,3**	**58,7**	**78**
GAPDH	Glyceraldehyde-3-phosphate dehydrogenase	**79,7**	**93**	**156,3**
HSPA8	Heat shock cognate 71 kDa protein	**54,3**	**57,3**	**104,3**
ACTG1	Actin, cytoplasmic 2	**128**	**99**	**115**
ANXA2	Annexin A2	**90**	**51,3**	**31,7**
CD9	CD9 antigen	**24,7**	**17,7**	**4**
PKM	Pyruvate kinase PKM	**82,7**	**134,7**	**103,7**
HSP90AA1	Heat shock protein HSP 90-alpha	**55**	**53**	**98**
ENO1	Alpha-enolase	**58,7**	**57**	**62,7**
ANXA5	Annexin A5	**31,3**	**12**	**29,3**
HSP90AB1	Heat shock protein HSP 90-beta	**31,7**	**26**	**54,3**
CD63	CD63 antigen	**0**	**0**	**4,7**
YWHAZ	14-3-3 protein zeta/delta	**35,3**	**17**	**21**
YWHAE	14-3-3 protein epsilon	**28**	**5,3**	**7**
EEF1A1	Elongation factor 1-alpha 1	**24,3**	**32,7**	**32,7**
PGK1	Phosphoglycerate kinase 1	**52,3**	**79,7**	**72,3**
CLTC	Clathrin heavy chain 1	**89**	**98,3**	**129**
PPIA	Peptidyl-prolyl cis-trans isomerase A	**33,3**	**27**	**37,7**
SDCBP	Syntenin-1	**30**	**32,3**	**61**
ALDOA	Fructose-bisphosphate aldolase A	**37,7**	**20,3**	**21**
EEF2	Elongation factor 2	**52**	**69**	**71,3**
ALB	Serum albumin	**21,7**	**9,7**	**5,7**
TPI1	Triosephosphate isomerase	**35,3**	**28**	**26,7**
VCP	Transitional endoplasmic reticulum ATPase	**14,3**	**15,3**	**17,3**
CFL1	Cofilin-1	**19,3**	**9,3**	**12**
MSN	Moesin	**93,3**	**60,3**	**60**
ATP1A1	Sodium/potassium-transporting ATPase subunit alpha-1	**69**	**32**	**31**
PRDX1	Peroxiredoxin-1	**13,3**	**14,7**	**17**
MYH9	Myosin-9	**142,3**	**60,3**	**89,7**
EZR	Ezrin	**34,7**	**41,3**	**9**
