# Supplementary material for: Cancer cell line-specific protein profiles in extracellular vesicles identified by proteomics
Source: PLoS One. 2020 Sep 4;15(9):e0238591. doi: 10.1371/journal.pone.0238591 (PMC7473518; doi:10.1371/journal.pone.0238591)
Supplement: S3 Table — (PDF) [file pone.0238591.s003.pdf]

Supplementary table 1:

EVs isolated from cell culture supernatants of E10, BxPC3, and H3, contain proteins shown to modulate tumor and metastatic environment.

			Cell Line (Spectral counts)		
Gene name	Protein description	Function	E10	BxPC3	H3
ADAM10	S-100 calcium binding protein A10	Contributes to cell migration and proliferation	**12,7**	**5,7**	**0**
ALCAM	CD166 ligand	Cell adhesion molecule	**21,7**	**17**	**0**
CDC42	Cell division control protein 42 homolog	RegulateS the dynamic organization of the cytoskeleton and membrane trafficking for physiologic processes, such as cell proliferation, motility, polarity, cytokinesis and cell growth	**15,3**	**14**	**7,7**
CSPG4	Chondroitin sulfate proteoglycan 4	Involved in cell proliferation and migration; may participate in melanoma cells invasion properties.	**34**	**4**	**96,3**
CTNNA1	Catenin alpha-1	Involved in cell adhesion; may play a role in cell differentiation	**68,3**	**26,7**	**25,7**
EGFR	Epidermal growth factor receptor	Activates at least 4 major downstream signaling cascades including the RAS-RAF-MEK-ERK, PI3 kinase-AKT, PLCgamma-PKC and STATs modules; Promotes cancer cell proliferation	**158,3**	**26,30**	**1,5**
FN1	Fibronectin	Involved in cell adhesion, cell motility, opsonization, wound healing, and maintenance of cell shape	**99,3**	**12**	**137,3**
ICAM	Intercellular adhesion molecule	Angiogenesis	**55**	**9,3**	**2,3**
ITGA2	Integrin, alpha 2	Member of the integrin adhesion receptors family; promotes intracellular signaling and tumor cell proliferation, migration and survival; when forming a heterodimer alpha2beta1 is crucial for angiogenesis.	**57**	**15**	**2**
ITGA3	Integrin alpha-3	Member of the integrin adhesion receptors family; promotes intracellular signaling and tumor cell proliferation, migration and survival; when forming the heterodimer alpha3beta1 increases migration of colon cancer cells	**58,7**	**15,7**	**17,7**
ITGA5	Integrin, alpha 5	Member of the integrin adhesion receptors family; promotes intracellular signaling and tumor cell proliferation, migration and survival; the heterodimer alpha5beta1 is involved in the increased migration of colon cancer cells.	**31**	**5,7**	**0**
ITGA6	Integrin alpha-6	Member of the integrin adhesion receptors family; promotes intracellular signaling and tumor cell proliferation, migration and survival; α6ß4 and α6ß1 heterodimers mediate cancer cell motility and metastasis	**66,3**	**31,7**	**0**
ITGB1	Integrin beta-1	Member of the integrin adhesion receptors family; Dimerizes with many other member of the integrin family, thus is involved in processes such as cell adhesion, cell motility and angiogenesis, and matrix degradation processes, promoting cell invasion.	**68,3**	**19,3**	**25,3**
ITGB4	Integrin, beta 4	Member of the integrin adhesion receptors family; promotes intracellular signaling and tumor cell proliferation, migration and survival; required for the regulation of keratinocyte polarity and motility.	**140,7**	**65**	**0**
LEG 1	Galectin 1	Immunosuppresive	**23,3**	**7,7**	**16,7**
LOXL2	Lysyl oxidase homolog 2	Involved in EMT and subsequent EMT induction; may play a role in tumor progression; inhibits keratinocyte differentiation	**20,3**	**7,3**	**3,3**
LOXL3	Lysyl oxidase homolog 3	Invasion, metastatic potential	**0**	**0**	**39,7**

MFGE8	Lactadherin	Angiogenesis MFG-E8 Drives Melanoma Growth by Stimulating Mesenchymal Stromal Cell–Induced Angiogenesis and M2 Polarization of Tumor-Associated Macrophages ; Extracellular vesicles including exosomes are mediators of signal transduction: are they protective or pathogenic?	**47,3**	**29,7**	**60,7**
MIF	Macrophage migration inhibitory factor	Inflammation (Exosomes in inflammation and role as biomarkers)	**14**	**7**	**7,7**
Muc1	Mucin-1	Involved in cellular contact and signaling; Involved in the dysregulated proliferation of tumor cells	**11**	**35**	**0**
Muc4	Mucin-4	May play a role in tumor progression repressing apoptosis as opposed to proliferation; plays an important role in cell proliferation and differentiation of epithelial cells	**5,3**	**43,3**	**0**
Muc5AC	Mucin 5 AC	Escape of cancer cells from immunosurvaillance/immunosuppressive agent	**89,7**	**406,7**	**13,7**
Muc18	Cell surface glycoprotein MUC18	Role in cell adhesion, and in cohesion of the endothelial monolayer at intercellular junctions. May allow melanoma cells to interact with cellular elements of the vascular system	**4**	**0**	**14**
PMEL	Melanocyte protein PMEL	Could protect tumor cells from antibody mediated immunity.	**0**	**0**	**26,3**
S100A9	S-100 calcium binding protein A9	Involved in inflammatory events and the development of metastatic disease	**15,3**	**9,3**	**0**
S100A14	S-100 calcium binding protein A14	Modulates P53/TP53 protein levels, thus regulates cell survival and apoptosis; can promote cell proliferation or apoptosis.	**8,7**	**10**	**0**
THBS1	Thrombospondin-1	TSP1 regulates diverse processes such as adhesion, invasion, migration, proliferation, apoptosis, immunity response and treatment response by interacting with multiple ligands	**15,3**	**53**	**0**
TIMP-1	Metalloproteinase inhibitor 1	Metalloproteinase inhibitor promotion of growth and survival of different transformed and tumoral cells	**8,7**	**11,7**	**3,7**
TINAGL1	Tubulointerstitial nephritis antigen-like	Cell Adhesion molecule	**52,7**	**58**	**2**
TNC	Tenascin C	Extracellular matrix protein; in tumors, stimulates angiogenesis by elongation, migration and sprouting of endothelial cells	**63**	**0**	**51**
VIM	Vimentin	Maintains cell integrity; overexpression is frequently associated with increased migratory/invasive capacity of cancer cells; Canonical marker of EMT	**0**	**0**	**46,7**
