# Supplementary material for: Cancer cell line-specific protein profiles in extracellular vesicles identified by proteomics
Source: PLoS One. 2020 Sep 4;15(9):e0238591. doi: 10.1371/journal.pone.0238591 (PMC7473518; doi:10.1371/journal.pone.0238591)
Supplement: S1 File — (PDF) [file pone.0238591.s004.pdf]

Supporting Information S1

Sample preparation and mass spectroscopy

Following characterization, proteomic analysis was carried out using a total of 20 µg of protein for each of the preparations (n=3 for each of the cell lines). Each sample was precipitated by adding 4 volumes of ice-cold acetone, vortexed and incubated overnight at -20°C. Samples were centrifuged (Centrifuge 5415R, Eppendorf, Hamburg, Germany) at 16,000 g for 20 min at 4°C. The supernatant was discarded and the pellets resuspended in 50 µl of a 6 M urea (Bio-Rad, Oslo, Norway) and 100 mM ammonium bicarbonate solution (Sigma-Aldrich, Oslo, Norway) (pH 7.8). In order to carry out the reduction and alkylation of cysteines, 2.5 µl of 200 mM DTT in 100 mM Tris-HCl (pH 8) were added to the samples and incubated for 1 hour at 37°C. Then, 7.5 µl of a 200 mM iodoacetamide solution (Sigma-Aldrich, Oslo, Norway) were added and the samples were incubated for 1 hour at room temperature protected from light. Alkylation was quenched with the addition of 10 µl of a 200 mM DTT solution (Sigma-Aldrich, Oslo, Norway) and incubated for 1 hour at 37°C. Then, proteins in all samples were digested with 10 µg of trypsin Gold (Promega, Madison, WI, USA) and incubated for 16 hours at 37°C, followed by the addition of 5 µl of 50% formic acid to stop the reaction. Resulting peptides were purified using an OMIX C18-micro SPE (Agilent, Santa Clara, CA, USA), and dried using a Speed Vac concentrator (Concentrator Plus, Eppendorf, Hamburg, Germany).

The tryptic peptides were resuspended in 10 µl of 0.1% formic acid/2% acetonitrile and analyzed on an Ultimate 3000 RSLCnano-UHPLC system connected to a Q Exactive mass spectrometer (Thermo Fisher Scientific, Bremen, Germany) equipped with a nano-electrospray ion source. For liquid chromatography separation, an Acclaim PepMap 100 column (C18, 2 µm beads, 100 Å, 75 µm inner diameter, 50 cm length) (Dionex, Sunnyvale CA, USA) was used. A flow rate of 300 nL/min was employed with a solvent gradient of 4-35% B in 60 min. Solvent A was 0.1% formic acid and solvent B was 0.1% formic acid/90% acetonitrile. The mass spectrometer was operated in the data-dependent mode to automatically switch between MS and MS/MS acquisition. Survey full scan MS spectra (from m/z 400 to 2,000) were acquired with the resolution R = 70,000 at m/z 200, after accumulation to a target of 1e5. The maximum allowed ion accumulation times were 60 ms. The method used allowed sequential isolation of up to the ten most intense ions, depending on

signal intensity (intensity threshold 1.7e4), for fragmentation using higher-energy collisional induced dissociation (HCD) at a target value of 1e5 charges, NCE 28, and a resolution R = 17,500. Target ions already selected for MS/MS were dynamically excluded for 30 sec. The isolation window was m/z = 2 without offset. For accurate mass measurements, the lock mass option was enabled in MS mode.
